# Success of the Companion

**Published:** 1872-03

**Authors:** 


### SUCCESS OF THE COMPANION.

We are gratified to state that our subscription-list is being increased rapidly; as almost every medical gentleman seeing the practical character of THE COMPANION, becomes a subscriber at once. The success of THE GEORGIA MEDICAL COMPANION *is unprecedented in the history of medical journals of the South.* From the very start, it established itself permanently in the esteem of the true professional men of the country, and is now, by their kindness, and love of principle, the largest circulated medical journal of its age, on the continent.

We wish to make it the best known and most widely circulated monthy in the country, and therefore, ask our friends, everywhere, to forward us the names of their medical friends whom they think would like to subscribe for a journal of its character.

